# Le retour d’« une seule santé » et la santé mondiale : ne reproduisons pas les mêmes erreurs

**DOI:** 10.48327/mtsi.v2i3.2022.255

**Published:** 2022-07-18

**Authors:** Valéry RIDDE, Étienne GUILLARD, Adama FAYE

**Affiliations:** 1 Université Paris Cité, IRD, INSERM, CEPED, Paris, France; 2 SOLTHIS, Solidarité thérapeutique et initiatives pour la santé, Paris, France; 3 ISED, Institut de santé et développement, Université Cheikh Anta Diop, Dakar, Sénégal

**Keywords:** Une seule santé, Mondialisation, Santé mondiale, One Health, Globalization, Global health

## Abstract

La pandémie liée à la COVID-19 a fait redécouvrir le concept d’« une seule santé » et l'idée que l'animal, l’être humain et l'environnement sont intimement liés. Ce concept n'est pourtant pas nouveau, mais il reste labile, ce qui contribue à créer une certaine confusion. Dans la pratique, les actions de terrain manquent encore cruellement et « une seule santé » ne parvient pas à intégrer les trois dimensions. Cet éditorial vise ainsi à partager six défis que devra relever la mise en oeuvre de l'approche « une seule santé » pour éviter les écueils d'autres initiatives de santé mondiale. Ainsi, les programmes dédiés à une seule santé ne pourront être pertinents et pérennes sans impliquer activement les communautés. En outre, ce déploiement implique une indispensable décolonisation de la santé, c'est-à-dire une remise en cause de la manière dont les programmes sont gouvernés, financés, formulés, mis en oeuvre et évalués, avec et pour les personnes et pays concernés. Elle ne pourra se faire sans s'attaquer aux inégalités sociales de santé et aux enjeux de pouvoir. Cette approche pousse à interroger les modèles d'exploitation des ressources tant agricoles que naturelles. Penser « une seule santé » implique de penser les problématiques et les interventions dans une perspective tant intersectorielle, inclusive et participative qu'interdisciplinaire, sinon transdisciplinaire et d'appréhender la complexité qui en résulte. Enfin, il conviendra de prendre en compte l'utilisation des résultats des recherches pour construire les actions et les politiques publiques. Prendre en compte ces différents défis et s'inscrire dans une perspective systémique et interdisciplinaire ancrée dans des contextes locaux selon une démarche participative et inclusive nous paraît ainsi essentiel pour répondre de manière appropriée, pertinente et durable aux enjeux associés à « une seule santé ».

## Abstract (en)

The COVID-19 pandemic has led to a rediscovery of the concept of “One Health” and the idea that animals, humans and the environment are intimately linked. This is not a new concept, but it is still labile, contributing to inevitable confusion. There is still a lack of action on the ground, and “One Health” fails to integrate all three dimensions. This editorial aims to share six challenges for implementing the “One Health” approach in order to avoid the pitfalls of other global health initiatives. One Health programmes cannot be relevant and sustainable without the active involvement of communities. This deployment implies the necessary decolonisation of health, i.e. a rethinking of how programmes are governed, financed, formulated, implemented and evaluated, with and for the citizens and countries concerned. It cannot be done without addressing social inequalities in health and power issues. This approach leads to questioning the exploitation models of both agricultural and natural resources. Thinking about “One Health” implies thinking about issues and interventions from an intersectoral, inclusive and participatory perspective, from an interdisciplinary, if not transdisciplinary perspective, and understanding the resulting complexity. Finally, research findings should be taken into account to build public actions. Considering these different challenges and adopting a systemic and interdisciplinary perspective anchored in local contexts according to a participatory and inclusive approach thus seems essential to us to respond in an appropriate, relevant and sustainable manner to the issues associated with “One Health”.

## Introduction

Dans cette période pandémique, le monde redécouvre le concept d’« une seule santé » [11]. Si nous nous réjouissons *a priori* de cette nouvelle mise en avant d'une approche essentielle pour améliorer la santé des populations humaines et animales dans des écosystèmes sains, nous souhaitons montrer dans cet éditorial qu'il est important de ne pas oublier certains principes essentiels, que l'approche actuelle de la santé mondiale a encore trop souvent mis de côté.

## Les Concepts et Leur Foisonnement

La pandémie que nous vivons maintenant depuis plus de deux ans a fait resurgir l'idée que l'animal, l’être humain et l'environnement sont intimement liés, cette triade pouvant définir simplement le concept d’« une seule santé » [22]. Bien évidemment, tout cela n'est pas nouveau pour celles et ceux qui ont entendu parler de l'unification microbienne du monde à partir du XIV^e^ siècle, parfaitement analysée par Le Roy Ladurie ou Gélinas [10]. De même, sans revenir sur les travaux de Rudolf Virchow au XIX^e^ siècle, les experts de la santé se souviendront du concept d’« une seule médecine » qui évoquait alors les interactions entre les humains et les animaux [22]. Depuis les années 1980, la promotion de la santé a quant à elle mit en exergue l'importance d'appréhender la santé à travers ses différents déterminants, parmi lesquels les environnements et milieux de vie figurent clairement. Les illustrations de cette interdépendance sont désormais nombreuses, y compris lorsque les communautés sont comprises comme ces milieux de vie.

Pourtant, sur le terrain, les acteurs de la santé animale et ceux de la santé humaine peinent encore à se rencontrer, se parler et collaborer, sans compter les faibles ressources dont disposent les systèmes de santé animale et le manque de cadre formel pour favoriser la collaboration. Par exemple, une recherche présentée au dernier colloque de la Société française de santé publique montre la quasi-absence de collaboration entre les vétérinaires et les médecins en France, pays qui ne manque pourtant pas de moyens [14]. Au Sénégal, dans le district de Kaffrine, pour une population de 260 000 habitants sont présents 93 professionnels de santé humaine contre seulement 9 professionnels de santé animale, soit 10 fois moins. Pourtant, une étude montre que les seconds ont 8 fois plus de chances de disposer de suffisamment de connaissances pour lutter contre la rage que les premiers [2]. La collaboration est donc essentielle à la santé des populations, humaines comme animales.

Aujourd'hui, lorsqu'on étudie les projets de développement, les documents de politique publique et qu'on écoute les discours de responsables des instances de santé mondiale, on est relativement surpris par la quantité phénoménale de concepts qui sont mobilisés pour évoquer des domaines relativement proches [1]. Ainsi, certains évoquent, et nous tentons de traduire de l'anglais tous ces concepts sans leur donner de chronologie d'apparition: une seule santé, une seule médecine, éco-santé, un seul monde, santé mondiale, médecine tropicale, santé internationale, santé durable, santé planétaire, santé unique, un seul monde une seule santé, santé publique vétérinaire, santé dans les systèmes socio-écologiques, etc. Tous ces concepts disposent d'experts et de revues savantes dédiées, tentant pour certains d’être présents et visibles pour chercher à créer un champ, voire une discipline spécifique. Rien que pour le concept d’« une seule santé », Ribeiro et ses collègues ont repéré en 2019 pas moins de six définitions différentes dans leur revue systématique de 56 articles, dont seulement quatre concernent les pays d'Afrique subsaharienne [7]. Malgré plusieurs tentatives de définition [17], cette prolifération de termes et de concepts similaires, mais sensiblement différents, a pour conséquence de créer une certaine confusion pour les acteurs de terrain. Ils n'en perçoivent pas les nuances ou l'intérêt face aux problématiques auxquelles ils font face au quotidien. Au-delà de cette multiplication des concepts se cachent aussi des visions distinctes, plus ou moins anthropocentrées. Ainsi, pour certains, le pilier de « santé environnementale » est appréhendé comme la santé de l'humain impactée par les environnements et non de la santé même de ces environnements et des écosystèmes associés, comme le soulignent d'ailleurs Lainé et Morand [12] en appelant à une approche plus qu'humaine.

## Mais Parle-T-On Vraiment de Concepts?

Contrairement à un inventaire à la Prévert, cette liste absolument non exhaustive traduit bien le foisonnement de concepts tous inter-reliés, mais aussi notre difficulté commune, mondiale, à les différencier, à les comprendre, à les utiliser, et surtout à les mettre en pratique. Selon Daigneault et Jacob, un concept doit être défini au regard de ses trois dimensions que sont le sens, le terme et le référent. De plus, « *seul un concept construit et opérationnalisé de manière adéquate peut contribuer a? une production scientifique cumulative de qualité* » [6]. C'est ce que certains nomment la maturité d'un concept. À nos yeux, si celui d’« une seule santé » l'est depuis longtemps, mettant au jour le besoin d'une approche transdisciplinaire et intersectorielle de la santé humaine, animale et environnementale, c'est encore loin d’être le cas pour celui de santé globale, également traduit par « santé mondiale ». En effet, depuis la médecine coloniale dont certains effets néfastes associés à ses pratiques coercitives (au Sénégal, on se rappellera de la gestion désastreuse de la peste et du choléra durant l’époque coloniale [8], ou plus récemment des débats sur l'essai du vaccin contre la méningite de 2007 [19]) se sont de nouveau révélés dans les approches contemporaines de santé publique pour lutter contre la pandémie de COVID-19 à travers le monde [5], il semble que les équipes qui tentent de déconstruire le concept de santé mondiale éprouvent des difficultés. Le concept de santé mondiale est très récent, et il a été mis en avant pour les besoins d'acteurs nord-américains et européens afin de qualifier leurs interventions et leurs enseignements de santé publique hors de leurs frontières nationales [20]. Le concept de santé internationale ayant du mal à se départir de son histoire coloniale, celui de santé mondiale tente de prendre de la distance, sans vraiment convaincre cependant.

## Profiter de L'opportunité Pandémique Pour Agir, Vraiment

Si nous évoquons cette question, c'est justement parce que nous pensons que l'urgence de santé mondiale, pour reprendre une expression à la mode, est en partie, mais pas seulement, la lutte contre cette pandémie. Mais cela ne doit pas se concrétiser à n'importe quel prix et n'importe comment. Il faudrait certainement profiter de cette opportunité pour remettre en question la manière dont nous travaillons collectivement. Si les approches d’« une seule santé » et de la « santé mondiale » sont complémentaires et non contradictoires, il ne faudrait pas que le renouveau de la première reproduise les erreurs de la mise en avant de la seconde. En effet, comme l'ont noté Morand et ses collègues, « *One Health ne parvient pas, en pratique, a? ve?ritablement inte?grer les trois dimensions me?dicale, ve?te?rinaire et e?cologique* » [13]. Pour la rendre plus concrète, il conviendrait d’éviter des approches trop génériques, souvent hors sol, pour appréhender des problématiques plus spécifiques (maladies infectieuses émergentes, résistances aux antimicrobiens, pollutions et contaminations, etc.) à des niveaux locaux où les trois composantes humains/animaux/environnements sont directement connectées. Cela impliquerait un ancrage de la santé communautaire et territoriale qui permettrait de rééquilibrer le positionnement encore trop institutionnel, voire trop intellectuel.

Ainsi, nous présentons très rapidement les moyens que nous pensons absolument essentiel de ne pas oublier collectivement dans la mobilisation que nous allons certainement voir du concept « une seule santé » dans les prochaines années, fusse-til de santé mondiale. Une liste relativement exhaustive des défis de cette approche a été présentée par d'autres [7] et nous nous concentrons sur les six que nous estimons les plus importants.

Avant tout, il est essentiel de voir concrétiser ce retour à une seule santé dans le contexte de la santé mondiale. Les bibliothèques regorgent de livres et de réflexions sur ces concepts tandis que **les politiques, les programmes et les actions de terrain manquent** encore cruellement [11], notamment en Afrique subsaharienne. Tout comme le discours sur la santé pour tous à l'an 2000 ou celui de la couverture universelle en santé ne se sont jamais vraiment concrétisés, notamment pour les plus pauvres, il ne faudrait pas que ce renouveau d’« une seule santé » reste au niveau des idées, des discours, des déclarations de principes et des intentions de financement. Appréhender « une seule santé », et ce qu'elle implique, pourrait ainsi être l'occasion de rappeler la nécessité d'agir sur des déterminants essentiels de la santé comme l'accès à une eau potable en continu, ou l'accès à des dispositifs d'assainissement et de gestion des déchets, si toutefois ces responsabilités sont assumées par les États et non déléguées à des ONG au gré des projets de l'aide internationale. Il nous faudra financer et agir collectivement, vite, sérieusement et rigoureusement. Cela impliquera des investissements conséquents, d'autant qu'ils devront être intersectoriels et s'inscrire dans des temporalités longues pour voir des changements dans les environnements et les écosystèmes. Temporalité par ailleurs critique dans le cadre d’« une seule santé », pour laisser le temps d'apprendre à parler le même langage, à travailler collectivement, à comprendre les perceptions et les problématiques des autres. Un temps que les mécanismes de financement laissent rarement avec la recherche de résultats à court terme. La verticalité couramment observée dans les pratiques des grandes organisations internationales interroge quant à leur capacité à s'inscrire dans l'intersectorialité pourtant essentielle en matière d’équité. Il conviendra de rester vigilants à ne pas reproduire des dispositifs d'aide internationale qui sont devenus tellement bureaucratiques et complexes qu'ils nécessitent des experts (internationaux) de ces dispositifs, fragilisant encore plus les pays récipiendaires.

### 1. Impliquer les communautés

Les politiques et programmes dédiés à « une seule santé » ne pourront être pertinents et pérennes sans **impliquer activement les communautés concernées** tant à l’échelle locale qu’à un niveau de gouvernance plus large [3]. Sur ces aspects, les initiatives de santé mondiale qui ont impliqué les communautés en leur donnant notamment une place dans les mécanismes de gouvernance à différents niveaux sont riches d'enseignements. Toutefois, quel que soit le niveau de participation, il dépendra de l'appropriation de ces enjeux par ces acteurs communautaires et du développement de leur pouvoir d'agir en conséquence. Agir en ce sens pose des bases robustes pour contribuer à répondre à des enjeux émergents, qu'ils soient sanitaires, environnementaux, climatiques.

### 2. Décoloniser la santé

Ensuite, on ne peut faire l'impasse sur l'indispensable **décolonisation de la santé** [4] dont tout le monde parle mais qui peine à se concrétiser. Elle implique une remise en cause de la manière dont les programmes de santé sont gouvernés (par des instances et des personnes sans diversité et non représentatives des pays et populations concernées), financés (par l'aide internationale et ses priorités), formulés (avec des référentiels néolibéraux), mis en oeuvre et évalués (par des experts du Nord, des consultants internationaux) avec et pour les personnes et pays concernés. Dans un contexte où, au Sénégal par exemple, 85% des projets de recherche sur la COVID-19 ont été financés par l'extérieur tout comme 84% de la riposte à la pandémie [9] (au Burkina Faso également), les défis d'une indépendance et les risques que les recherches et les actions ne répondent pas aux besoins des populations nationales sont grands. Cette décolonisation devrait, non seulement s'affranchir du (post-)colonialisme, mais aussi « *du racisme, du sexisme, du capitalisme et d'autres -ismes nuisibles […] pour l’équité en santé* [4] ». Il nous faudra donc penser à décoloniser nos pensées mais aussi nos financements, nos formations et nos actions. Les autorités des pays concernés devraient pouvoir prendre plus conscience du besoin de leurs engagements pour plus de financements nationaux et d'actions locales.

### 3. S'attaquer aux inégalités sociales

Après, il faut s'attaquer aux **inégalités sociales de santé et aux enjeux de pouvoir** qui sont au coeur de nos sociétés, et influencent indubitablement nos interventions de terrain et nos pratiques de recherche. Pourtant, ces inégalités sont le plus souvent ignorées par les personnes qui formulent les interventions de lutte contre les épidémies [15] alors que les connaissances pour s'y attaquer existent. La pandémie a foncièrement exacerbé les inégalités en Europe et ailleurs dans le monde. Il faudra certainement s’éloigner des tentatives de stigmatisation des individus, comme nous l'avons encore vu dans les instruments mobilisés pour lutter contre la pandémie [5], et plutôt agir sur les systèmes sociaux et les structures en place. En effet, « *lutter contre les inégalités en santé, ce n'est pas vouloir aider les plus vulnérables, c'est s'interroger sur le rôle des privilégiés et remettre en cause les structures sociales qui créent les privilèges et les oppressions* [16] ».

### 4. Interroger les modèles d'exploitation des ressources

Par ailleurs, « une seule santé » doit nécessairement interroger nos sociétés sur les modèles d'exploitation des ressources, tant agricoles que naturelles, et des conséquences que cela entraîne. Ces choix ont des implications économiques et sociales qui peuvent avoir des effets sur le niveau de vie d'une population et ainsi aggraver ou atténuer les inégalités sociales de santé. La lutte contre Ebola en Guinée a été entravée par la volonté de l’État et ses partenaires commerciaux internationaux de ne pas mettre en péril son exploitation minière [18]. Dans le cas des déforestations intensives, sans autres solutions adaptées et financièrement acceptables, on ne peut pas demander à une population de ne pas couper les forêts et de ne pas consommer du gibier de brousse. Il en est de même pour l'usage des pesticides. Sans l'ajout de ces produits, la production de certaines cultures peut être fortement réduite. Or, les tarifs sur les marchés internationaux s'avèrent tellement bas que ces rendements sont totalement insuffisants pour permettre aux producteurs de vivre décemment. Outre les dimensions économiques, ces exemples soulignent les dimensions éminemment politiques d’« une seule santé », encore largement omises eu égard aux orientations en matière de développement, d'exploitation des ressources et d'aménagement des territoires. Cela interroge par ailleurs les capacités de régulation et de réglementation notamment des opérateurs privés à une double échelle, d'une part nationale – mais délicate car concerne des systèmes de santé de pays fragiles – et d'autre part internationale – avec une mise en application d'autant plus difficile qu'elle implique des multinationales ou des modes de production que certains ne souhaitent pas remettre en cause.

### 5. Penser des approches intersectorielles

Puis, il devient nécessaire de penser nos approches dans une perspective tant **intersectorielle, inclusive et participative qu'interdisciplinaire, sinon transdisciplinaire**. Pourtant, nos formations académiques, nos revues scientifiques, nos politiques publiques, nos actions de terrain, nos financements sont encore tous très loin de l’être [11]. Nous savons qu'il sera impossible de lutter contre le SARS-CoV-2 et toutes autres calamités en cours ou à venir, sans cette intersectorialité, sans cette vision interdisciplinaire qui tient compte de la complexité des enjeux et d'une vision systémique. Un vaccin seul ou une réflexion uniquement biomédicale sur un problème de santé, comme c'est encore trop souvent le cas, ne permettra pas de le résoudre. Cette recherche de solutions simples, ces fameuses « magic bullets » que l'on pourrait transposer partout, demeure malheureusement trop souvent utilisée en santé publique et mondiale. L'approche « une seule santé » a aussi cette tendance réductrice qu'il faut combattre. Pour reprendre l'expression de Chimamanda Ngozi Adichie concernant les « single stories » que nous avons récemment utilisées pour analyser la lutte contre la pandémie qui fait fi des enjeux de la **complexité**, l’écrivaine nigériane affirme: « *L'histoire unique crée des stéréotypes, et le problème des stéréotypes n'est pas qu'ils sont faux, mais qu'ils sont incomplets. Ils font qu'une histoire devient la seule histoire* [21]. » Il faudra absolument lutter contre la construction de cette vision unique d'une santé unique, même si elle figure dans le nom même, tant pour appréhender les problèmes de santé que pour développer et mettre en oeuvre les politiques publiques et interventions de terrain de manière inclusive et participative. La complexité est inhérente à l'approche d’« une seule santé » et devra être prise en compte pour s'adapter dans des contextes spécifiques en tenant compte de problématiques territoriales. Dans ce domaine, comme d'ailleurs en santé publique et santé mondiale plus globalement, il s'agira moins de trouver UNE solution qui puisse être transposée que de déployer un processus de diagnostic et d'adaptation participatif et inclusif à ces contextes et s'adaptant à la complexité.

### 6. Utiliser les résultats des recherches

Enfin, une dimension encore trop peu prise en compte est bien celle de **l'utilisation des résultats des recherches**, oubliant la science de l'utilisation de la science. Par exemple, le Groupe d'experts intergouvernemental sur l’évolution du climat (GIEC) se plaint, à juste titre, du manque de prise en compte de leurs travaux par les responsables politiques. Mais ils procurent à ces décideurs des rapports de synthèse (de leurs rapports scientifiques de plusieurs centaines de pages) de plus de 50 à 100 pages et d'un niveau de technicité inaccessible. En outre, nous constatons, d'une part, que trop peu d'interventions de terrain tiennent compte de l’état des connaissances scientifiques pour construire leurs actions et choisir leurs instruments en les adaptant aux contextes nationaux et locaux. D'autre part, encore moins d'activités sont planifiées pour renforcer les chances que les résultats des études soient utiles et utilisés par les décideurs politiques mais aussi par les communautés et les personnes concernées. Il devient essentiel de mieux former l'ensemble des acteurs concernés mais aussi, sinon surtout, de planifier des activités spécifiques dont nous savons qu'elles peuvent plus facilement contribuer à l'utilisation de la recherche par les milieux de pratiques et politiques.

## Conclusion

Dans cet éditorial, nous avons souhaité mettre l'accent sur six principaux défis que l'approche « une seule santé », dont on entendra certainement encore parler pendant longtemps, devra nécessairement tenir compte si les personnes qui l'animent ne souhaitent pas reproduire les mêmes erreurs que celles qui soutiennent la santé mondiale. Pour l'avenir, le défi principal est de penser l'approche « une seule santé » selon une perspective systémique et interdisciplinaire ancrée dans des contextes locaux selon une démarche participative et inclusive. Pour y arriver, il nous faudra collectivement changer nos modes de formations académiques et professionnelles en s’éloignant des approches biomédicales de la santé pour se rapprocher des enseignements interdisciplinaires et soucieux de la justice épistémique. Nos politiques publiques et nos interventions de terrain devront partir des besoins locaux, impliquer toutes les personnes concernées et expertises interdisciplinaires pour proposer des solutions adaptées et respectueuses des humains, des animaux et de l'environnement. L'implication du secteur privé (industries et entreprises) devrait devenir centrale pour qu'il puisse montrer son implication sociale réelle (et non uniquement discursif) à améliorer la santé de la planète et de ses habitants avec la collaboration des États et du secteur public. Vaste programme sans lequel le concept d’« une seule santé » sera aussi inopérant que celui de la santé pour tous en l'an 2000.

## Liens D'intérêts

Les auteurs ne déclarent aucun lien d'intérêt.

## Contribution des Auteurs

Ce texte est une adaptation longue d'une conférence prononcée par Valéry Ridde lors du Forum Galien Afrique de décembre 2020 (que nous remercions) et dont le contenu a été largement remanié et amélioré par les contributions d'Etienne Guillard et Adama Faye. Tous les auteurs ont contribué à l'ensemble de l'article.
